# Endometriosis and eating disorders: epidemiology, shared neurobiology, and clinical implications

**DOI:** 10.1007/s00404-026-08325-2

**Published:** 2026-01-21

**Authors:** Stefano Di Michele, Chiara Camoglio, Pierluigi Chieppa, Giosuè Giordano Incognito, Alessandro Caiazzo, Alessia Cabras, Federica Picci, Stefano Angioni

**Affiliations:** 1 Department of Surgical Sciences, Division of Obstetrics and Gynecology, University of Cagliari, Cagliari, Italy; 2 Department of Surgical Sciences, Gynecology and Obstetrics, 1, A.O.U., City of Health and Science of Turin, S. Anna Hospital, Turin, Italy; 3 Department of General Surgery and Medical Surgical Specialties, University of Catania, 95123 Catania, Italy

**Keywords:** Endometriosis, Eating disorders, Emotional eating, Binge eating disorder, Diet, Neurobiology

## Abstract

Growing evidence suggests that women with endometriosis may be particularly vulnerable to disordered eating behaviors (DEBs) and clinically defined eating disorders (EDs). This narrative review aims at integrating and critically analyzing the current evidence regarding the relationship between endometriosis and EDs, as well as highlighting the psychosocial and neurobiological vulnerabilities of women with endometriosis to DEBs. A large-scale genetic study showed a nearly threefold increase in the odds of EDs in women with endometriosis, and a significant genetic correlation. Although the prevalence of formal ED diagnoses appears low in small clinical samples, DEBs such as emotional eating, binge tendencies, and maladaptive dietary restriction, are common and strongly associated with pain intensity, and borderline BMI. Psychological factors, including body image disturbance, heightened self-criticism, emotional dysregulation, and the need for control further contribute to the vulnerability to EDs. At the biological level, the dysregulation of leptin, endocannabinoids, dopamine, brain-derived neurotrophic factor, and inflammatory cytokines, molecules involved in both appetite regulation and some aspects of the pathophysiology of endometriosis, suggests overlapping neuroimmune pathways that may link endometriosis to DEBs and EDs. Clinical management must, therefore, integrate screening for DEBs, supervised and personalized dietary counseling, balanced exercise prescription, and psychological interventions targeting pain coping, emotion regulation, and body image. A multidimensional, biopsychosocial framework is essential to prevent the onset or exacerbation of EDs in women with endometriosis.

**Supplementary Information:**

The online version contains supplementary material available at 10.1007/s00404-026-08325-2.

## Introduction

Endometriosis is a chronic, debilitating disease associated with pelvic pain and infertility, which affects an estimated 5–10% of reproductive-aged women [1]. Despite its prevalence, diagnosis is frequently delayed, misdiagnosis is common, and effective treatment is often postponed [2]. Endometriosis is characterized by the presence of endometrial tissue outside the uterus. Different forms have been described: superficial or peritoneal, deep or sub-peritoneal, and ovarian endometriomas [2]. However, increasing evidence suggests that endometriosis is not just a pelvic disease, but rather a chronic systemic condition in which ectopic lesions create a proinflammatory microenvironment and promote angiogenesis, neuroangiogenesis, and nociceptive sensitization [3]. Therefore, a comprehensive understanding of the disease is essential, as it contributes to disrupted metabolism, pain sensitization, mood disturbances, and several psychiatric comorbidities, including depression, anxiety, and eating disorders (EDs) [3, 4].

EDs result from a complex interaction of psychological vulnerability, biological predisposition, and sociocultural pressures that together distort body image and eating behavior. Recent decades have seen an approximately 25% increase in global prevalence, especially during adolescence, which constitutes the peak risk period [5]. Lifetime prevalence is estimated at around 8.6% in women compared to 4.1% in man, and nearly one in five women will experience an ED by age 40 [6]. Disturbed attitudes toward weight, body shape, and eating are central to their development and maintenance, and they often manifest overlapping syndromes [7]. DSM-5 and ICD-11 provide diagnostic frameworks for feeding and EDs, which include anorexia nervosa (AN), bulimia nervosa (BN), binge-eating disorder (BED), and other specified presentations [8]. In AN, an intense fear of weight gain leads to persistent dietary restriction, severe undernutrition, endocrine dysfunction, and multi-organ complications, with mortality rates among the highest of all psychiatric disorders [9]. Conversely, BN is characterized by recurrent binge-eating episodes followed by compensatory behaviors (such as self-induced vomiting, laxative misuse, fasting, or excessive exercise), reflecting a destructive cycle of loss of control and guilt [10]. By contrast, BED retains the binge component but lacks compensatory behaviors and is frequently associated with overweight, obesity, and metabolic disturbances [11]. In avoidant-restrictive food intake disorder (ARFID) and related conditions, food restriction is motivated by sensory sensitivity, fear of aversive consequences, or inherently low appetite, culminating in nutritional deficiencies and psychosocial impairment [12]. Finally, although less common and primarily documented in pediatric populations, pica and rumination disorder may also occur in women. Psychiatric comorbidity is common: over 70% of women with an ED meet criteria for at least one additional psychiatric condition, most often mood or anxiety disorders, obsessive–compulsive traits, substance-use disorders, or personality disorders, which contribute to greater symptom severity, lower psychosocial functioning, and poorer treatment outcomes [13]. Women with eating disorders face additional sex-specific risks such as amenorrhea, bone demineralization, infertility, endometriosis, and obstetric complications, all of which underscore the profound systemic burden of these illnesses. The clinical presentation of EDs is often unclear and confounding, and their recognition requires an integrated understanding of psychiatric, metabolic, and reproductive health [4, 6]. Moreover, maladaptive eating behaviors and unhealthy weight control strategies that do not meet the formal DSM-5 criteria for an ED, broadly defined as disordered eating behaviors (DEBs), are common and represent significant risk factors for the development of full-blown EDs [8].

**Fig. 1:**
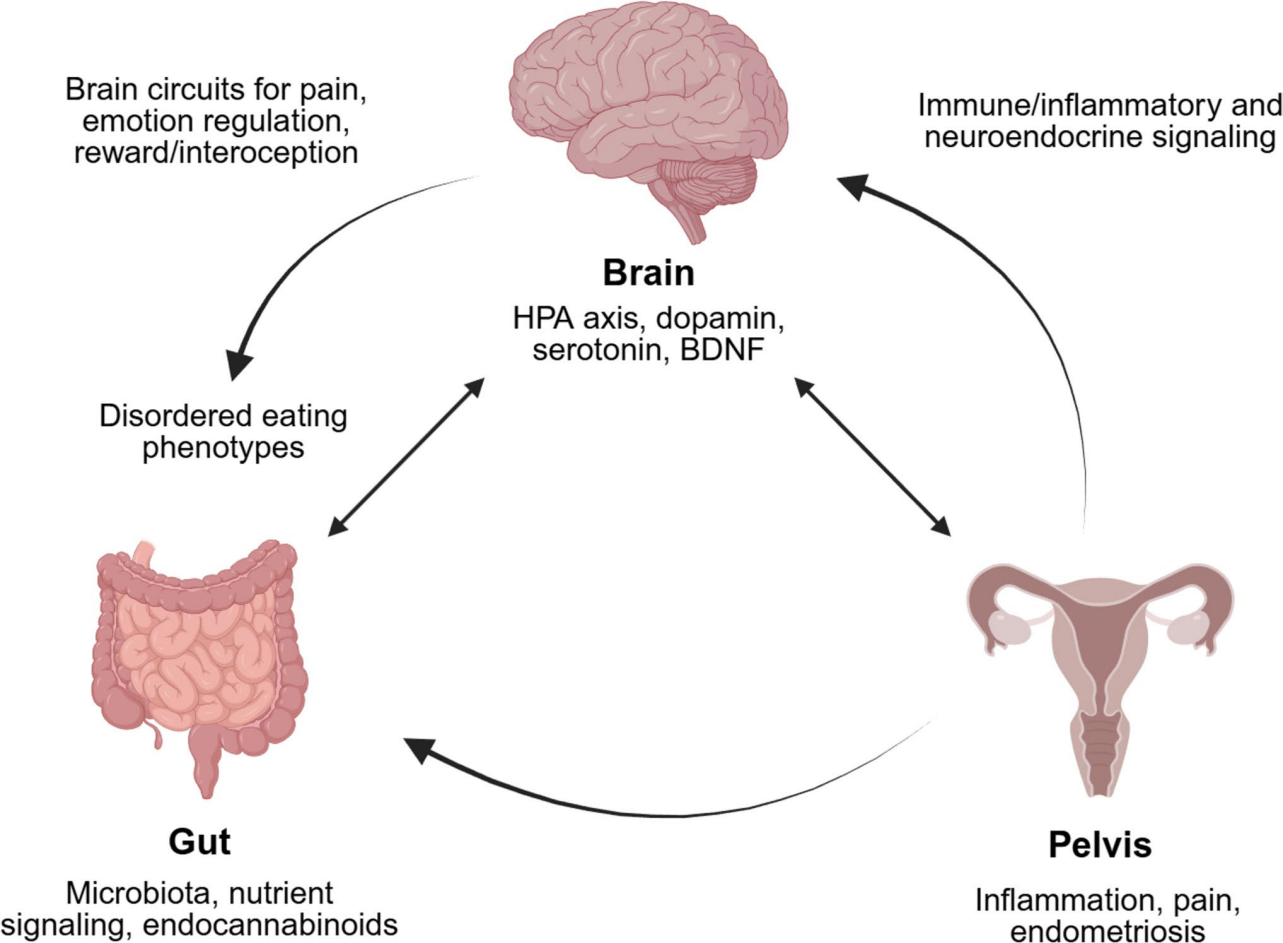
Bidirectional interactions between the brain, gut, and pelvis in modulating inflammation, pain, and endometriosis: an integrated network of neuroendocrine, immune and metabolic signaling also contributing to disordered eating phenotypes. Created in BioRender. Camoglio, C. (2026) https://BioRender.com/y3jwmzt

In this review, EDs refer to clinically diagnosed eating disorders within DSM/ICD frameworks. In contrast, questionnaire-based tools are reported to serve as screening measures for ED risk or DEBs rather than as diagnostic substitutes. Emerging evidence suggests a bidirectional relationship between endometriosis and disordered eating, in which chronic pain, systemic inflammation, and altered hypothalamic–pituitary–adrenal (HPA) axis activity may foster maladaptive eating behaviors. At the same time, nutritional imbalance, stress-related neuroendocrine dysregulation, and metabolic disturbances can, in turn, exacerbate endometriosis-associated inflammation and pain [14]. This complex interplay is framed within the concept of the brain-gut-pelvis axis, highlighting the multidirectional neuroimmune and neuroendocrine communication linking emotional regulation, gastrointestinal (GI) function, and pelvic pain syndromes [15] (Fig. 1).

The present review seeks to consolidate existing knowledge on the intersection between endometriosis and EDs by examining their epidemiological coexistence, shared neuroimmune and neuroendocrine alterations, and clinical consequences. Moreover, it aims to identify specific biological and psychosocial vulnerabilities in women with endometriosis that may increase their susceptibility to develop eating pathology, providing an integrated perspective on prevention and management.

## Methods

This work was designed to integrate and critically analyze the current literature on the relationship between endometriosis and EDs. The review followed a structured, domain-focused approach rather than a systematic one, given the emerging and heterogeneous nature of the evidence.

### Search strategy and study selection

PubMed/MEDLINE, Scopus, and Web of Science were searched from inception to October 31, 2025. Full database-specific search strings (epidemiological/clinical and molecular queries) are provided in Online Resource 1. Reference lists of eligible articles and relevant reviews were hand-searched to identify additional records.

Eligibility criteria were intentionally broad due to the emerging and heterogeneous nature of the topic. We included studies involving women/girls with endometriosis (self-reported, surgically confirmed and/or clinically/imaging diagnosed) that reported: clinically defined EDs according to DSM/ICD frameworks or clinical diagnosis, and/or DEBs assessed by validated screening instruments or questionnaires (e.g., SCOFF, EAT-26, EDE-Q, BES, YEOQ), and/or psychological correlates (e.g., body image constructs) or biological mediators plausibly linked to eating behavior (e.g., leptin, endocannabinoids, dopamine/serotonin pathways, BDNF, inflammatory cytokines). Epidemiological, clinical, genetic, and qualitative studies were considered; narrative/systematic reviews were used to contextualize emerging themes.

PC, AC, FP, and AC screened epidemiological/clinical studies; CC screened molecular studies. Any disagreement was resolved by discussion with SDM and SA.

### Data extraction and synthesis

The included studies were grouped thematically into the following domains: epidemiology and comorbidity patterns between endometriosis and EDs; psychological and behavioral correlates, including DEBs, emotional eating attitudes (EEAs), and body image disturbance; biological and neuroendocrine mediators potentially linking the two conditions; and clinical and therapeutic implications encompassing nutritional and lifestyle interventions.

A qualitative synthesis was then performed across these domains. The evidence was analyzed in a critical and integrative manner, emphasizing areas of convergence, inconsistency, and research gaps. When possible, biological findings (e.g., alterations in leptin, endocannabinoids, dopamine, serotonin, and brain-derived neurotrophic factor (BDNF)) were correlated with behavioral and clinical observations to generate a multidimensional interpretative model.

## Emerging connections between endometriosis and EDs

In recent years, there has been growing attention to the potential association between endometriosis and EDs [4, 16–18] (Table 1). The most compelling evidence for an association between the two conditions comes from a large-scale genetic association study [4]. Women with endometriosis had a significantly higher probability of developing eating disorders (EDs) compared to the general population (OR: 2.94; 95%CI 1.96–4.41), independently of several confounding factors such as BMI, chronic pain, irritable bowel syndrome (IBS), and comorbid psychiatric conditions. In addition, significant genetic correlation was found between endometriosis and EDs, as well as other psychiatric disorders, such as depression and anxiety, indicating possible shared pleiotropy [4]. In line with these findings, smaller studies have reported further evidence characterizing the association between endometriosis and EDs. In the ENDONUT pilot study [16], 19 of 54 (35.2%) women with endometriosis scored positive on the SCOFF-F questionnaire, which represents the validated French adaptation of the original SCOFF instrument [19], a five-item questionnaire according to which two or more positive responses suggest possible ED risk and warrant further clinical assessment, indicating a higher likelihood of DEBs/ED symptomatology. On the other hand, a preliminary cross-sectional investigation in Italy reported that only 1 out of 30 patients (~ 3.33%) screened positive for clinically significant binge-eating symptomatology (consistent with BED features) based on the Eating Disorder Examination-Questionnaire EDE-Q, which provides a Global score and four subscales (Restraint, Eating Concern, Shape Concern, Weight Concern) [20] and the Binge Eating Scale (BES) cut-offs [21], used to assess binge-eating severity. However, mean scores for DEBs and EEAs were significantly associated with clinical risk conditions, such as increased BMI and moderate/severe intermenstrual pain. In particular, emotional overeating due to pain (“physical pain” in the Yale Emotional Overeating Questionnaire (YEOQ) scale, which captures the frequency of overeating in response to specific emotions, such as sadness, anxiety, anger and boredom [22]), was positively correlated with intermenstrual pain intensity, suggesting that food intake may represent a maladaptive coping strategy overcome pain and negative emotions. Therefore, although the prevalence of formal diagnoses was low (~ 3.3%), DEBs and maladaptive emotional attitudes toward food were present at significant levels, particularly in association with chronic pain and borderline BMI. Indeed, women with a BMI greater than 22.4 scored significantly higher on the Eating Attitudes Test-26 (EAT-26) bulimia and food preoccupation subscales [23] and on the BES. This suggests that, in endometriosis, weight fluctuations may result from DEBs/EEAs rather than being exclusively attributable to metabolic factors or medical treatments [17, 24]. The link between chronic pain and eating is consistent: emotional eating can be a response to physical pain, in line with neuroendocrine models of “dysfunctional hunger” [25]. However, the small sample size, the lack of a control group, and the exclusion of women with a comorbid diagnosis of depression limit clinical significance and potentially under- or overestimate the association between endometriosis and EDs [17]. A more recent qualitative study investigating disordered eating in 179 women with endometriosis reported high levels of eating disorder psychopathology, increased self-criticism, cognitive rigidity regarding body image, and body dissatisfaction [18]. Frequently reported issues among participants included perceiving the body as unfamiliar or disrupted, conceptualizing food as threatening or aversive, and reporting a diminished sense of bodily well-being and identity. All these factors are consistent with the complex biopsychosocial burden of endometriosis and may represent potential predisposing factors for the development of a full-blown ED.

**Table 1:** Studies addressing EDs in women with endometriosis

Study	Study design	Study setting	Participants	Sample size	Instruments	Key findings	Confounder adjustment	Limitations
Aupetit et al., 2022	Cross-sectional study (pilot)	Rouen University Hospital, France	Women with histologically confirmed EM from CIRENDO cohort	*N* = 54	SCOFF-F; EAT-26	19 women with EM (35.2%) had a positive SCOFF-F score, 10 (18.5%) had positive EAT-26 score, suggesting possible ED risk. Positive SCOFF-F score was associated with increased anxiety and depression scores	None	Small sample size; Lack of control group; All included patients were from the same hospital; Half of the patients had digestive endometriosis Recent EM diagnosis (less than 4 years)
Koller et al., 2023	Genetic association study	International, multicenter, population-based	Women of European descent with self-reported or ICD-10 diagnosis of EM from the UK Biobank cohort combined with genome-wide statistics from large biobanks and consortia	EM = 8276; Controls = 194,000	Logistic regression (phenotype association); Genome-wide association analysis; SCORE; LDSC; Mendelian randomization	EM was associated with increased odds of EDs (OR: 2.94; 95%CI: 1.96–4.41), depression (OR: 3.61; 95%CI: 3.32–3.92), and anxiety (OR: 2.61; 95%CI: 2.30–2.97). Significant genetic correlation was found between EM and EDs (rg = 0.61, *p* = 0.02), depression and anxiety	Age; BMI; Chronic pain-related phenotypes; IBS; Socioeconomic status; Psychiatric comorbidities	Limited statistical power; Possible detection bias; Limited sample diversity
Panariello et al., 2023	Cross-sectional study (preliminary)	Specialist outpatient clinic for EM in Bologna, Italy	Women with a documented clinical diagnosis of EM	*N* = 30	EAT-26; EDE-Q; BES; DEAS; YEOQ; NRS	1 woman (3.33%) scored positively for both EDE-Q and BES, suggesting a possible diagnosis of BED. Subthreshold scores in all instruments, suggestive of DEBs and EEAs, were associated with increased BMI and pain	Analyses were stratified based on BMI and NRS score	Small sample size; Lack of control group; Exclusion of patients with psychiatric disorders; BMI was derived from self-reported declarations of weight and height
Pellizzer et al., 2025	Mixed-method observational study (cross-sectional)	Community-based, Australia	Women with EM recruited from EM support groups on social media	*N* = 179	BI-AAQ; EDE-Q7; DASS21; FSCRS	EDE-Q7 scores indicate high levels of DEBs, ED psychopathology and body image concerns. BI-AAQ, DASS21 and FSCRS scores indicate high prevalence of body dissatisfaction, negative affect, and self-criticism	None	Possible selection and detection biases; Lack of internal control group; Only 6 of the 7 items of the EDE-Q7 were presented to participants; Descriptive data on EM condition were not included; Limited sample diversity

*EM* endometriosis; *ED* eating disorder; *EAT-26* Eating Attitudes Test-26; *SCORE* Scalable Genetic Correlation Estimator; *LDSC* Linkage Disequilibrium Score; Regression; *BMI* Body Mass Index; *IBS* Irritable Bowel Syndrome; *EDE-Q* Eating Disorder Examination-Questionnaire; *BES* Binge Eating Scale; *DEAS* Disordered Eating Attitude Scale; *YEOQ* Yale Emotional Overeating Questionnaire; *NRS* Numeric Rating Scale for EM-associated pain; *BI-AAQ* Body Image-Acceptance and Action Questionnaire; *DASS21* Depression Anxiety and Stress Scales 21; *FSCRS* Forms of Self-Criticising/Attacking and Self-Reassuring Scale; *BED* binge-eating disorder; *EEAs* emotional eating attitudes

## Predisposing factors for the development of EDs in women with endometriosis

The association between endometriosis and EDs suggests the existence of shared etiological pathways that extend beyond the impact of chronic pain alone [4]. Furthermore, the presence of a previous diagnosis of EDs has been linked to a higher probability of subsequently receiving a diagnosis of endometriosis [26]. Other factors, such as diet and lifestyle, might contribute to their co-occurrence. Dietary management in endometriosis patients occupies an ambivalent space, offering potential symptom relief while simultaneously introducing risks for developing disordered eating patterns [18]. Recent findings suggest that adjusting diet and lifestyle, particularly by incorporating vitamins, minerals, and anti-inflammatory foods, can significantly help manage symptoms and improve quality of life [27, 28]. Medical nutrition therapy is emerging as a valid, non-pharmacological approach to managing symptoms by modulating systemic inflammation, oxidative stress, and hormonal imbalance inherent to the disease’s pathophysiology [28, 29]. In particular, the Mediterranean diet (MedDiet) is recommended as an optimal long-term nutritional strategy for normoweight women with endometriosis, especially those dealing with chronic pelvic pain or GI symptoms [29]. However, when followed without appropriate supervision, restrictive diets may pose significant metabolic and long-term health risks. Prolonged adherence to highly restrictive plans, such as the low-FODMAP diet, has been discouraged due to the potential for nutrient deficiencies and adverse effects on gut microbiota composition and diversity [30]. The widespread availability of unverified dietary advice is concerning, as much information, especially online or in popular media, lacks evidence-based support. This underscores the need for individualized, professionally supervised dietary counseling to ensure both safety and efficacy in managing endometriosis [27]. Additionally, adherence to highly restrictive diets may promote obsessive tendencies. The requisite emphasis on dietary control often entails increased vigilance and continuous cognitive monitoring of food intake. Such stringent evaluation of ingredients and imposed dietary restrictions can generate considerable psychological distress and feelings of guilt [18]. In the ENDOBELLY study, following restrictive diets, participants reported binge eating, comfort eating, and overeating in response to frustration or sadness caused by the illness and its impact on their identity [18].

In this context, physical activity and exercise have been proposed as potential complementary interventions. Their effects, however, are inherently ambivalent. Although supervised exercise offers significant therapeutic benefits for symptom management and psychological well-being, it may also contribute to the development of DEBs in women with endometriosis, particularly in the context of elevated psychopathology, body dissatisfaction, and a pathological need for control, transforming physical activity into compulsive and dysfunctional behavior due to the psychological vulnerabilities present in this population [31–34]. In this context, exercise, like dietary practices, may be used to exert control over one’s life, counteracting feelings of powerlessness and bodily betrayal induced by chronic illness [18, 34, 35]. Excessive exercise can thus function as a compensatory behavior, analogous to patterns observed in AN and BN, shifting from therapeutic activity to a symptom of underlying psychopathology [18, 34].

Paradoxically, chronic pain may drive the opposite behavioral response, indirectly increasing DEBs’ risk [17]. Pain can trigger fear-avoidant behaviors, leading to reduced physical activity and subsequent deconditioning [36]. This inactivity may contribute to increased BMI, which is negatively associated with physical HRQoL, which includes physical and mental well-being of individuals and is positively correlated with heightened eating-related psychopathology and emotional eating [4, 17, 18, 37].

Body image disturbance is highly prevalent in people with endometriosis, acting as a critical risk factor for EDs [35]. In women affected by endometriosis, body dissatisfaction has been linked to numerous factors, including pain, infertility, weight gain, surgical scars, lack of body familiarity, loss of sense of self, and changed functioning [38]. Studies reveal that high percentages of participants with endometriosis report that their shape influenced how they judged themselves as a person or expressed high body dissatisfaction [18, 38, 39]. This severe psychological distress often manifests as feelings of loss of control and powerlessness [24, 39]. The onset of EDs and DEBs in individuals with endometriosis is profoundly influenced by psychological mechanisms rooted in chronic illness and emotional dysregulation [22, 40]. The primary mechanism is the pursuit of control. Facing a chronic, systemic disease like endometriosis, the sensation of loss of control drives some individuals toward strict regulation of their body or diet as a means "to control their lives in some way". This control is amplified by a diminished self-image stemming from concerns about appearance and functionality [18]. Indeed, women with endometriosis exhibit high levels of eating disorder psychopathology, often defined by the overevaluation of shape and weight, and severe pain acts as a primary stressor, strongly correlating with EEA [17, 18]. Emotional eating may be an adaptive coping strategy to manage negative affective states, including sadness, loneliness, anxiety, anger, and physical pain [22, 40, 41]. Overall, the overlap of psychological and behavioral factors, including the need for control, body dissatisfaction, emotional dysregulation, and the adoption of maladaptive coping strategies, highlights the vulnerability of women with endometriosis to develop DEBs and eventually EDs.

## Neuromodulators of eating behavior in endometriosis: a link with predisposition to eating disorders

Eating behavior results from the interplay between homeostatic and hedonic signals in the central nervous system. Peripheral hormones and peptides coming from the GI tract and adipose tissue integrate with vagal inputs and reach the hypothalamus to regulate energy homeostasis, while neurotransmitters and neuromodulators involved in reward processes and pleasurable responses to food regulate hedonic eating [42]. Disruptions of this finely tuned mechanism lead to aberrant eating patterns and characterize the pathogenesis of EDs [43].

**Fig. 2:**
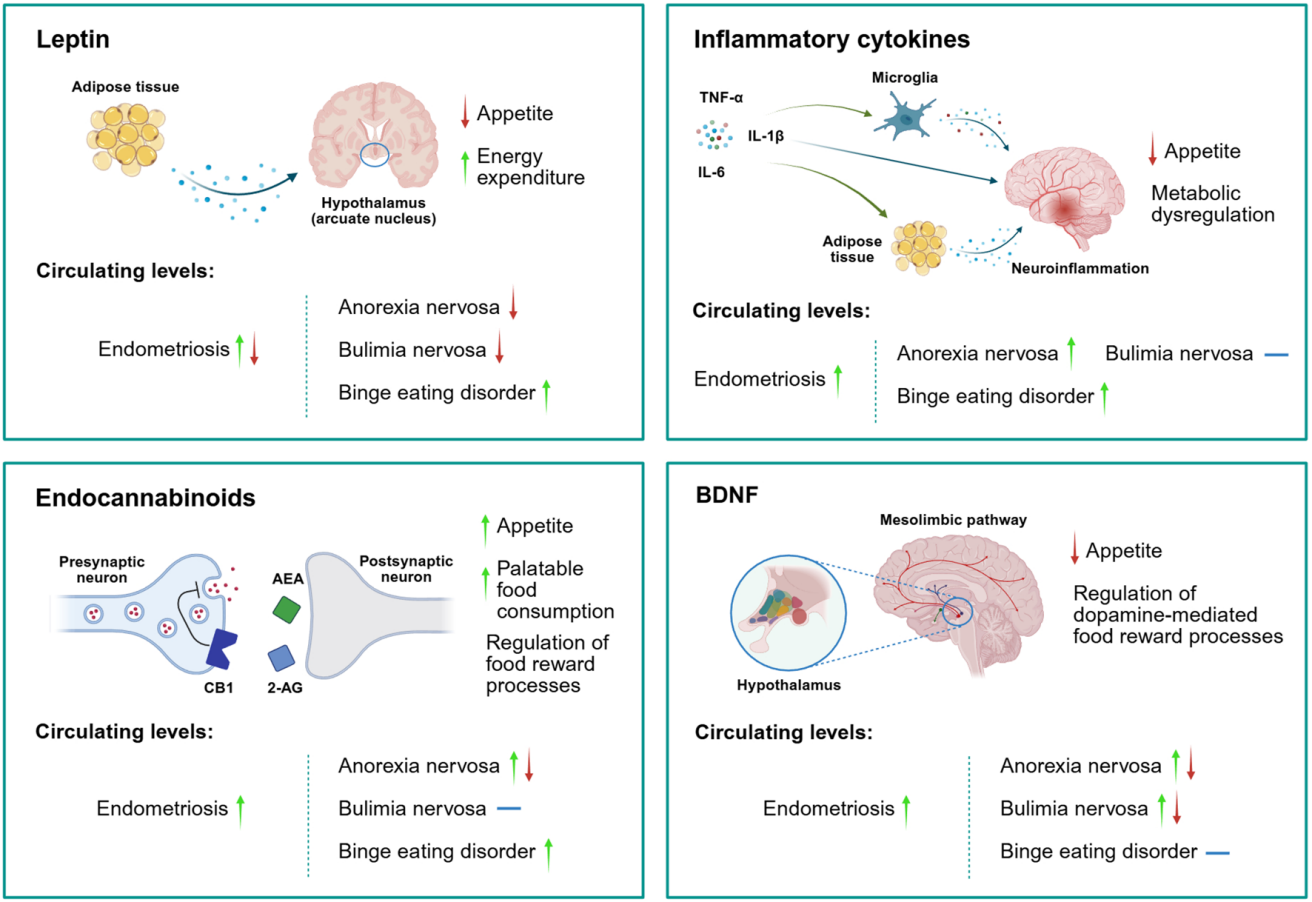
Neuromodulators of eating behavior in endometriosis and EDs. The arrows adjacent to each disorder indicate reported trends in circulating levels of the corresponding neuromodulators, measured in serum or plasma. *Leptin* Leptin is secreted by the adipose tissue and acts on the hypothalamus to suppress appetite and promote energy expenditure. Both increased and decreased circulating leptin levels have been observed in endometriosis, while leptin levels were reportedly increased in BED, and decreased in AN and BN. *Inflammatory cytokines* Peripheral pro-inflammatory cytokines can suppress appetite and induce metabolic alterations, either through direct action on hypothalamic neurons or by activating microglia, thereby causing neuroinflammation. Pro-inflammatory cytokines can also increase leptin production. Increased levels of pro-inflammatory cytokines have been observed in endometriosis, AN, and BED, but not BN. *Endocannabinoids* Endocannabinoids increase appetite and promote the consumption of palatable food through the activation of CB1 in the hypothalamus, and are involved in hedonic eating and the regulation of food reward. Endocannabinoid levels were increased in endometriosis and BED, and bidirectional alterations were reported in AN. *BDNF* BDNF participates in the homeostatic regulation of eating behavior by acting on specialized neurons in several hypothalamic nuclei. In addition, it regulates hedonic eating, food reward, and the motivation to eat by acting on the mesolimbic dopaminergic pathway. Increased circulating BDNF levels were observed in endometriosis, and bidirectional alterations were reported in EDs. NOTE: The available literature on this topic is still limited; trends may differ according to the biological compartment, disease stage, patient-specific characteristics, and study design or methodological approaches. Created in BioRender. Camoglio, C. (2026) https://BioRender.com/uyi51f2

Notably, modulators of eating behavior, such as leptin, endocannabinoids, and dopamine, have also been implicated in aspects of the pathophysiology of endometriosis. As with EDs, altered levels of these molecules were observed in women with endometriosis, further supporting a correlation between the two conditions [44, 45] (Fig. 2).

Leptin, also known as the satiety hormone, is an adipokine secreted by adipose tissue in response to body fat stores and is crucial for the homeostatic regulation of appetite and body weight. Under conditions of positive energy balance, leptin is produced, crosses the blood–brain barrier, and reaches the anorexigenic neurons in the arcuate nucleus of the hypothalamus, triggering a cascade that reduces appetite and increases energy expenditure. In addition, this hormone is involved in the regulation of metabolism, inflammatory processes, angiogenesis, and reproduction [46]. Increased leptin levels were correlated with leptin resistance, food addiction, and emotional eating [47, 48]. Conversely, lower levels have been reported in AN and BN [25]. Evidence of altered leptin in endometriosis is conflicting. Increased leptin levels were reported in follicular and peritoneal fluid, while reports on serum levels are inconsistent [44]. A recent study reported increased plasma leptin levels [49], and a systematic review reported an elevated leptin-to-BMI ratio in women with endometriosis, with significantly lower leptin levels in advanced stages [50]. In the context of endometriosis, leptin may contribute to the pathogenesis and progression of endometriotic lesions by influencing local angiogenesis and inflammation [51], and altered levels may also contribute to the impaired eating habits of many women with this condition.

Endocannabinoids are a class of molecules derived from arachidonic acid that bind to the cannabinoid receptors. These molecules are produced in response to changes in homeostasis and the external environment, and exert critical regulatory functions involved in appetite, metabolism, immunity and inflammation, nociception, and reproduction [25]. Although the exact mechanism remains unclear, the two most famous endocannabinoids, N-arachidonoylethanolamine (AEA) and 2-arachidonoylglycerol (2-AG), can stimulate hunger and food intake via cannabinoid receptor 1 (CB1) [25]. AEA levels are reportedly increased in women with AN and BED [52], and are thought to mediate the hedonic response to food, further sustaining binge-eating behavior [53]. Similarly, women with endometriosis exhibit elevated circulating levels of 2-AG and AEA, as well as decreased endometrial CB1 expression. In addition, AEA levels were higher in women with severe dyspareunia and dysmenorrhea [45]. Alteration of the endocannabinoid system in endometriosis likely contributes to the inability to properly manage pain and maintain the proinflammatory environment that sustains this condition [54]. Still, it might also increase the susceptibility of these women to EDs [25].

Dopamine and serotonin are essential regulators of hedonic eating, motivation to eat, rewarding properties of food, and mood. In particular, dopamine stimulates the consumption of pleasurable food and vice versa, while serotonin levels are inversely correlated with food intake, with low serotonin promoting food consumption [55]. Alterations in both the serotonergic and dopaminergic pathways are frequently reported in EDs and have been suggested as potential predisposing factors [56]. Despite limited evidence reporting alterations in serotonin and dopamine levels in endometriosis, given the frequent co-occurrence of this condition with depression and mood disorders, alterations in these neuronal pathways may be common in endometriosis [4, 26, 57]. Moreover, some evidence indicates that dopamine receptors may be involved in angiogenesis and the development of endometriotic lesions [58], and a study reported that a genetic polymorphism in the dopamine receptor D2 is associated with severe endometriosis [59]. Notably, another polymorphism of the same gene was linked to the genetic susceptibility to EDs [60].

The neurotrophin BDNF, which plays a crucial role in synaptic plasticity, learning, neuronal differentiation, and survival, is thought to regulate hedonic eating through its interplay with dopaminergic and serotonergic pathways and is involved in EDs due to its anorexigenic effect [25]. Circulating levels of BDNF were shown to be significantly increased in women with endometriosis [61], potentially influencing several aspects of eating behavior.

In addition, factors such as systemic inflammation and alterations in oxidative stress mechanisms, as well as chronic stress, which can activate the hypothalamic–pituitary–adrenal axis, are known to exacerbate and promote endometriosis symptoms [62, 63]. Notably, these factors are also deeply involved in the regulation of eating behavior and the pathophysiology of EDs [64], making them a risk factor that should not be overlooked. Interestingly, inflammatory cytokines, such as IL-1β, TNF-α, and IL-6, which are involved in eating behavior through their interactions with hypothalamic neurons and other brain systems [65], were elevated in the serum of women with endometriosis [66]. Similar patterns were observed in women with AN and obesity [67, 68].

Overall, the association between endometriosis and EDs appears to be supported by both psycho-social factors and shared underlying neurobiological mechanisms. This susceptibility should be taken into account when prescribing diets to manage symptoms in women with endometriosis, as excessive dieting can trigger the onset of EDs in predisposed individuals [32].

Taken together, alterations in leptin, endocannabinoids, dopamine, serotonin, and BDNF create a neuroendocrine environment marked by impaired reward processing, disrupted appetite regulation, and increased pain sensitivity. These mechanisms may explain why women with endometriosis are prone to disordered eating.

## Critical discussion and clinical perspectives

The converging evidence reviewed here supports a multidimensional vulnerability model in which chronic inflammation, pain-related neuroplasticity, and altered reward/interoceptive processing interact with psychosocial stressors to shape eating behavior in women with endometriosis. The association between endometriosis and EDs is consistent across studies [4, 16–18], and biological data suggest partially overlapping neurobiological pathways with EDs. Yet, critical methodological gaps remain: most studies are cross-sectional, often small, and variably adjusted for confounding factors, limiting causal inference. Experimental work in animal models indicates brain and behavioral changes linked to endometriosis and stress [63], and Mendelian randomization studies support a bidirectional association between endometriosis and mood disorders. However, causal evidence for EDs itself is still preliminary [4, 26].

Systematic screening is warranted in high-risk phenotypes. Women presenting with chronic pelvic pain, significant GI symptoms, marked body image concerns, or weight fluctuation should be routinely screened for disordered eating. Brief, validated tools (e.g., SCOFF [19]) can be used in gynecologic settings as first-line screens, followed, when positive or equivocal, by more granular instruments such as EAT-26 [23], BES [21], or YEOQ to capture emotional overeating patterns [22]. The ENDONUT pilot and subsequent cross-sectional data indicate that subthreshold DEBs and EEAs are common even when formal ED diagnoses are infrequent, and correlate with pain intensity and borderline BMI [16, 17]. Early identification of DEBs may therefore function as a preventive strategy, potentially mitigating symptom escalation and metabolic sequelae [22, 69].

Nutrition should be personalized, evidence-based, and monitored over time. Dietary changes can improve pain and quality of life through anti-inflammatory and metabolic effects, with the MedDiet representing a reasonable long-term option in normoweight women, especially in those with GI comorbidity [27–29]. In contrast, highly restrictive regimens (e.g., prolonged low-FODMAP outside supervised phases, or unverified eliminations from social media) carry risks of nutrient deficiency, microbiota perturbation, and induction/worsening of DEBs [30]. Given the illness-induced drive for control and the cognitive vigilance required by strict diets, clinicians should frame nutrition as symptom-management rather than identity-defining, set finite trial periods with predefined outcomes, and involve a nutrition professional skilled in these conditions [18, 23].

Exercise prescription should balance analgesia and psychopathology risk. Supervised multimodal programs improve pain, sleep, fatigue, sexual function, mental health, and overall HRQoL [31, 34, 37]. However, in patients with body dissatisfaction, perfectionism, or high need for control, exercise can become compulsive and operate as a compensatory behavior akin to that seen in AN/BN [32, 34]. Practical safeguards include: graded plans co-designed with the patient, explicit non-weight-centric goals (pain, function, mood), scheduled rest days, and routine checks for guilt/distress when sessions are missed, signals of unhealthy exercise [31, 33].

Address emotion regulation and body image as core treatment targets. Emotional eating frequently serves to regulate negative affect and pain [17, 18, 22, 38, 40, 41]; difficulties in emotion regulation are strongly linked to worse pain and lower quality of life in endometriosis [40]. Embedding psychological interventions, cognitive-behavioral therapy-based pain coping, acceptance-based strategies, mindfulness, and body image work within gynecologic care can attenuate both pain amplification and maladaptive eating responses [39, 40, 70]. Given the high comorbidity with anxiety/depression [4, 26, 57], low-threshold referral pathways to mental health are essential.

Communicate the biology to reduce stigma and support adherence. Explaining to patients that neuroendocrine changes (e.g., leptin resistance, endocannabinoid signaling, stress-HPA activation, BDNF dynamics) may couple pain, mood, and eating urges [49–51, 61–63, 66] can legitimize symptoms, de-pathologize coping attempts, and increase engagement with non-restrictive nutritional and psychological plans.

### A pragmatic pathway for clinicians

At presentation, screen for DEBs/EEAs (SCOFF ± EAT-26/BES/YEOQ) and mood symptoms; flag red flags (rapid weight loss, syncope, purging, marked bradycardia) for urgent eating disorder referral. For most patients, combine:

- Analgesia/endometriosis management as ESHRE recommendation [33];
- Dietary counseling anchored to MedDiet principles with time-limited trials for gastrointestinal triggers [27–29];
- Exercise delivered as a supervised, function-oriented program [31];
- Psychological care focused on pain coping, emotion regulation, and body image [17, 18, 22, 38–40, 70].

Reevaluate at 6–12 weeks with jointly defined outcomes (pain, HRQoL, DEB indices) and adjust.

### Limitations

This narrative review synthesizes an emerging and heterogeneous literature; therefore, several limitations apply. First, included studies vary substantially in design, populations, and outcome measures, with frequent reliance on self-report questionnaires that screen for DEBs/ED risk rather than establish clinical diagnoses. Second, most available clinical studies are cross-sectional, small, and inconsistently adjusted for key confounders (e.g., BMI, pain severity, IBS, psychiatric comorbidity), limiting causal inference and increasing the risk of residual confounding. Third, molecular findings are often preliminary and not fully comparable across biological compartments, disease stage, and assay methodologies. The neuromodulators were selected based on established evidence of their role in the regulation of eating behavior and the pathophysiology of EDs, the possibility that other relevant molecules were not considered cannot be excluded.

### Future directions

Prospective cohorts are needed to chart temporal sequences between pain flares, mood changes, diet/exercise behaviors, and DEBs. Trials should test integrated interventions against usual care, with biomarkers, digital phenotyping of eating and activity, and neuroimaging of reward-pain circuits as mechanistic endpoints.

In summary, recognizing and managing disordered eating in endometriosis is not ancillary but central to comprehensive care. A biopsychosocial, multidisciplinary model that aligns nutrition, movement, pain science, and psychological therapies is both feasible and likely to yield superior outcomes for pain, function, and long-term health.

## Supplementary Information

Below is the link to the electronic supplementary material.

Supplementary file1 (DOCX 18 KB)

## Data Availability

No datasets were generated or analysed during the current study.
