# Supplementary material for: Endometriosis and eating disorders: epidemiology, shared neurobiology, and clinical implications
Source: Arch Gynecol Obstet. 2026 Jan 21;313(1):58. doi: 10.1007/s00404-026-08325-2 (PMC12823736; doi:10.1007/s00404-026-08325-2)
Supplement: Supplementary file 1 — Supplementary file1 (DOCX 18 KB) [file 404_2026_8325_MOESM1_ESM.docx]

| **Online resource 1:** Strings used for bibliographic search | | |
| --- | --- | --- |
| **Pubmed/MEDLINE** | *Epidemiological and clinical studies* | ("Endometriosis"[MeSH]  OR endometriosis[tiab])  AND  ("Feeding and Eating Disorders"[MeSH]  OR "eating disorder*"[tiab]  OR "disordered eating"[tiab]  OR "emotional eating"[tiab]  OR "body image"[tiab]) |
|  | *Molecular studies* | ("Endometriosis"[MeSH]  OR endometriosis[tiab])  AND  ("Leptin"[MeSH]  OR leptin[tiab]  OR "Endocannabinoids"[MeSH]  OR endocannabinoid*[tiab]  OR "Brain-Derived Neurotrophic Factor"[MeSH]  OR BDNF[tiab]  OR "Dopamine"[MeSH]  OR dopamine[tiab]  OR serotonin  OR "inflammatory cytokine*"[tiab]) |
| **Scopus** | *Epidemiological and clinical studies* | TITLE-ABS-KEY ( endometriosis )  AND  TITLE-ABS-KEY ("eating disorder*"  OR "disordered eating"  OR "emotional eating"  OR "body image") |
|  | *Molecular studies* | TITLE-ABS-KEY ( endometriosis )  AND  TITLE-ABS-KEY (leptin  OR endocannabinoid*  OR "brain-derived neurotrophic factor"  OR BDNF  OR dopamine  OR serotonin  OR "inflammatory cytokine*") |
| **Web of Science** | *Epidemiological and clinical studies* | TS=(endometriosis)  AND  TS=(eating disorder*  OR disordered eating  OR emotional eating  OR body image) |
|  | *Molecular studies* | TS=(endometriosis)  AND  TS=(leptin  OR endocannabinoid*  OR "brain-derived neurotrophic factor"  OR BDNF  OR dopamine  OR serotonin  OR inflammatory cytokine*) |
